# Omega-3 Supplementation Improves Isometric Strength But Not Muscle Anabolic and Catabolic Signaling in Response to Resistance Exercise in Healthy Older Adults

**DOI:** 10.1093/gerona/glaa309

**Published:** 2020-12-07

**Authors:** Sebastiaan Dalle, Evelien Van Roie, Charlotte Hiroux, Mathias Vanmunster, Walter Coudyzer, Frank Suhr, Stijn Bogaerts, Ruud Van Thienen, Katrien Koppo

**Affiliations:** 1 Exercise Physiology Research Group, Department of Movement Sciences, KU Leuven, Belgium; 2 Physical Activity, Sports and Health Research Group, Department of Movement Sciences, KU Leuven, Belgium; 3 Department of Morphology and Medical Imaging, Faculty of Medicine, Radiology Section, KU Leuven, Belgium; 4 Locomotor and Neurological Disorders, Department of Development and Regeneration, KU Leuven, Belgium; 5 Research Group for Neurorehabilitation, Department of Rehabilitation Sciences, KU Leuven, Belgium

**Keywords:** Aging, Inflammation, Muscle wasting, Resistance training, Sarcopenia

## Abstract

Old skeletal muscle exhibits decreased anabolic sensitivity, eventually contributing to muscle wasting. Besides anabolism, also muscle inflammation and catabolism are critical players in regulating the old skeletal muscle’s sensitivity. Omega-3 fatty acids (ω-3) are an interesting candidate to reverse anabolic insensitivity via anabolic actions. Yet, it remains unknown whether ω-3 also attenuates muscle inflammation and catabolism. The present study investigates the effect of ω-3 supplementation on muscle inflammation and metabolism (anabolism/catabolism) upon resistance exercise (RE). Twenty-three older adults (65–84 years; 8♀) were randomized to receive ω-3 (~3 g/d) or corn oil (placebo [PLAC]) and engaged in a 12-week RE program (3×/wk). Before and after intervention, muscle volume, strength, and systemic inflammation were assessed, and muscle biopsies were analyzed for markers of anabolism, catabolism, and inflammation. Isometric knee-extensor strength increased in ω-3 (+12.2%), but not in PLAC (−1.4%; *p*_interaction_ = .015), whereas leg press strength improved in both conditions (+27.1%; *p*_time_ < .001). RE, but not ω-3, decreased inflammatory (p65NF-κB) and catabolic (FOXO1, LC3b) markers, and improved muscle quality. Yet, muscle volume remained unaffected by RE and ω-3. Accordingly, muscle anabolism (mTORC1) and plasma C-reactive protein remained unchanged by RE and ω-3, whereas serum IL-6 tended to decrease in ω-3 (*p*_interaction_ = .07). These results show that, despite no changes in muscle volume, RE-induced gains in isometric strength can be further enhanced by ω-3. However, ω-3 did not improve RE-induced beneficial catabolic or inflammatory adaptations. Irrespective of muscle volume, gains in strength (primary criterion for sarcopenia) might be explained by changes in muscle quality due to muscle inflammatory or catabolic signaling.

Ageing is associated with systemic and local physiological adaptations that impair skeletal muscle metabolism and eventually induce loss in muscle functioning and muscle quantity or quality, referred to as sarcopenia. Currently, resistance exercise (RE) is considered the primary therapeutic strategy to attenuate the progression of age-related loss in muscle mass, strength, or functioning. However, compared to young adults, older adults (OAs) (~70 years) are less responsive to anabolic stimuli such as RE (1) and protein supplementation (2). Therefore, there is a need for strategies to overcome this age-related anabolic resistance.

There are several mechanisms that contribute to anabolic insensitivity in OAs, including inflammation (3), decreased blood flow and thus nutrient delivery to the muscle (4), insulin insensitivity (5), and muscle adiposity/lipotoxicity (6). Hence, attenuation of these factors is a promising strategy to maximize the anabolic potential of RE. Recently, there is a growing interest in the detrimental role of inflammation, both systemically and in the muscle tissue, on muscle functionality and metabolism (7). Inflammation might not only attenuate the anabolic mTORC1 pathway (8), it also stimulates muscle catabolism (9). This drives the muscle towards a negative protein balance, eventually contributing to muscle wasting. Hence, anti-inflammatory strategies might play a key role in combatting muscle wasting.

Indeed, nonsteroidal anti-inflammatory drugs (NSAIDs) improve the anabolic sensitivity in older rats (20 months) (10). Furthermore, among OAs (~85 years), NSAID users had a nearly 80% lower risk of being affected by sarcopenia (11). However, on the long term, NSAID use might cause adverse events such as gastrointestinal and cardiovascular injuries (12). Ω-3 polyunsaturated fatty acids (PUFAs) are an interesting alternative, given their anti-inflammatory and anabolic mechanisms of action (13). Ω-3 supplementation was shown to lower systemic levels of proinflammatory cytokines in overweight (14) and obese (15) adults, and in patient populations (16), but not consistently in (middle-)aged adults (50–95 years) (17–19). Besides their anti-inflammatory potential, ω-3 PUFAs might play a therapeutic role in the context of muscle ageing (13) due to stimulation of the mTORC1 pathway and concomitant muscle protein synthesis (20). Together, these observations have raised the interest among health practitioners to apply ω-3 PUFAs as a therapeutic strategy in combatting (age-related) muscle wasting. Furthermore, ω-3 treatment can also downregulate the catabolic ubiquitin-proteasome and autophagy pathways in rodent and muscle cell atrophy models such as sepsis (21), cancer cachexia (22), and starvation (23). However, it remains unclear whether ω-3 treatment in (older) humans also attenuates skeletal muscle catabolism, and whether this is regulated through muscle inflammation.

In OA, ω-3 is effective to improve muscular adaptations to anabolic interventions such as RE (13). Yet, the molecular mechanisms underlying these beneficial adaptations due to ω-3 are not understood. Therefore, the present study investigates the effect of ω-3 PUFAs supplementation on muscle molecular signaling in response to RE in OAs (>65 years). More specifically, we studied for the first time the effect of ω-3 on muscle markers of inflammation (eg, NF-κB, TNFα), autophagy (eg, LC3b, p62, beclin-1), and the ubiquitin-proteasome system (UPS; eg, FOXO, MuRF1, MAFbx), concomitantly with mTORC1 signaling, and measures of muscle strength and functional capacity. We hypothesize that ω-3 supplementation improves muscular adaptations to RE, that is, muscle strength and volume, at least partly through an upregulation of muscle anabolism and downregulation of muscle catabolism and inflammation.

## Method

### Participants

Twenty-three community-dwelling, nonsarcopenic OAs (65–83 years; 8♀) participated in the study and were informed in detail on the aim and the procedures of the study protocol, and signed a written informed consent (Supplementary Table 1). This study was approved by the Ethics Committee Research UZ/KU Leuven (S61809). Exclusion criteria included body mass index (kg/m^2^) <20 or >35, unstable body weight (2-kg change during the past 6 months), smoking, disease (cancer, liver, renal, musculoskeletal, neurodegenerative, or unstable cardiovascular dysfunctions), structural RE, ω-3 PUFA supplementation in the past year, and regular NSAID intake in the past 3 months. Participants were medically screened by a physician to check eligibility to engage in RE. Furthermore, participants were asked to maintain their habitual physical activity and diet during the intervention, and to limit their fatty fish consumption (salmon, mackerel, tuna, herring, trout, and sardines).

### Study Design

Participants were enrolled in a 14-week, placebo-controlled, double-blind study and randomized to a placebo (PLAC) or ω-3 PUFA (ω-3) supplementation condition. Randomization was performed by a researcher who was otherwise not involved in the study and conditions were matched for age, sex, inflammation status, and muscle strength. Subjects were supplemented for 14 weeks. After 2 weeks of priming with ω-3 or PLAC, participants engaged in a supervised 12-week RE program.

### Supplementation

Participants in the ω-3 condition ingested 3 times daily 1100 mg ω-3 PUFAs softgels (1020 mg ω-3; 410 mg DHA + 540 mg EPA; 4 mg vitamin E) (Vista-Life Pharma, Brussels, Belgium). In the PLAC group, participants daily ingested 3 iso-caloric, appearance-matched softgels (1100 mg corn oil; Vista-Life Pharma). Compliance was assessed via an itinerary and based on the remaining capsules at regular time points.

### Resistance Training

Prior to the RE protocol, participants were familiarized with training modalities, that is, leg press (Signature Series, Life Fitness, IL), leg extension (Optima Series), and standing calf raises with weight vests. The 12-week RE program involved 3 supervised sessions (~40 minutes) per week on nonconsecutive days. Each session started with a 10-minute cycle ergometer warm-up (Technogym, Gambettola, Italy) at self-selected resistance and 70–80 rpm. Next, lower extremity exercises were performed at a moderate speed, that is, 3 seconds for both the concentric and eccentric action. Between sets, 1- to 2-minute recovery time was provided. In the first 6 weeks of RE, 2 sets of 12–15 repetitions at ~70% of the 1-repetition maximum (1-RM) were performed, while in the last 6 weeks, 3 sets of 10–12 repetitions at ~80% 1-RM were performed. Participants were encouraged to complete the last set until failure. Resistance was adjusted if participants performed repetitions beyond the prescribed training zone to ensure maximal effort at the end of each exercise set to optimize muscular adaptations (24).

### Muscle Biopsy Procedure

On arrival at the lab and prior to the first biopsy, participants rested for 45 minutes. A needle biopsy (~150 mg) of the *m. vastus lateralis* was performed under local anesthesia (2% xylocaine, 1 mL subcutaneously) with a 5-mm Bergström-type needle. Four biopsies were taken, 2 at baseline (PRE) and 2 after completion of the 14-week intervention (POST). All biopsies were taken at least 72 hours after RE to exclude interference of acute exercise on muscle molecular signaling (25). The first biopsy (proximal needle orientation, both on the PRE- and POST-session) was taken in a fasted state and the second biopsy (distal needle orientation) was sampled 80 minutes after a standardized, protein-rich breakfast consisting of 4 toasts (4 × 22 g), jam (40 g), and a protein shake (34 g powder in 0.3 L H_2_O) (in total: 565.8 kcal; 38.2 g proteins of which 13 g essential amino acids; 4.1 g fats of which 0.5 g saturated; 93.2 g carbohydrates of which 25.1 g sugars; 3.3 g fibers; 1.4 g NaCl). The protein shake contained the following essential amino acids: 2.9 g l-leucine, 1.8 g l-isoleucine, 2.6 g l-lysine, 0.6 g l-methionine, 0.8 g l-phenylalanine, 2.0 g l-threonine, 1.2 g l-tryptophan, and 1.6 g l-valine. Part of the muscle sample was immediately frozen in liquid nitrogen and stored at −80°C for later analyses. The remaining part was mounted in embedding medium (Tissue-Tek OCT), frozen in precooled isopentane, and stored at −80°C until histochemical analyses were performed. We and others showed that 2 biopsies on the same day (proximal and distal needle orientation) do not affect the expression of markers of inflammation, protein synthesis, and breakdown (26,27).

Procedures for RNA extraction, reverse transcription, and real-time quantitative PCR analyses (primer sequences, see Supplementary Table 2), as well as protein extraction and western blot analyses are described in the Supplemental Material.

### Outcome Measures

#### Muscle strength

The 1-RM was measured on the leg press device as previously described (28). Shortly, participants started with a warm-up set of 8 repetitions at ~50% of the estimated 1-RM, followed by a set of 3 repetitions at ~70% of the estimated 1-RM. Subsequent lifts were single repetitions with progressively heavier resistances until failure. A 2-minute recovery period was provided between each attempt. The heaviest successful lift was determined as 1-RM. Participants were familiarized with the 1-RM leg press procedure. The 1-RM was assessed at baseline (PRE), after 6 weeks of RE, and after 12 weeks of RE (POST).

Maximal isometric strength of the knee extensors was tested on the Biodex Medical System 3 dynamometer (Shirley, NY). The test was performed with the right leg. Participants were seated on a backwardly inclined (5°) chair and secured with safety belts across the upper leg, the hips, and the shoulders. The rotational axis of the dynamometer was aligned with the transversal knee-joint axis and was attached to the tibia with a length-adjustable lever arm. The position of the rotational axis and the chair, as well as the length of the lever arm were identical at PRE- and POST-test. The isometric test consisted of 4 maximal bouts at a knee angle of 90°, with 20-second rest intervals in between. Isometric strength was determined as the highest peak torque (N∙m) during a 5-second leg extension. Throughout the test, participants were given verbal encouragement to assure maximal effort.

#### Muscle volume

A computed tomography scan (Somatom Force, Siemens Medical Solutions, Erlangen, Germany) was used to measure muscle volume of the left upper leg. Four 5-mm-thick axial images were obtained at the midpoint of the distance between the medial edge of the trochanter major and the intercondyloid fossa of the femur. Standard Hounsfield units (HU) ranges for muscle (0−100) were used to segment muscle tissue area, and corrections were made for bone marrow. The 4 slices were put together as one 20-mm-thick slice. Total muscle volume was determined with a software program developed at the University Hospital by one expert radiologist who was blinded to the group allocation.

Calf circumference was measured at a plane perpendicular to the long axis of the calf while the participant was sitting on chair with a 90° angle in the knee of the dominant leg and the foot flat on the floor.

#### Muscle quality

Muscle quality, that is, the association between strength and mass (29), of the upper leg was calculated as the knee-extensor isometric peak torque divided by muscle volume of the upper leg, expressed in N∙m/cm^3^. Higher HU of skeletal muscle tissue on the CT scans also refers to a higher muscle quality.

#### Functional performance

Functional performance was evaluated with several tests: 5-repetition chair sit-to-stand test (5STS), 30-second chair sit-to-stand test (30STS), timed up-and-go test (TUG), maximal gait speed test (MGS), and handgrip strength test (HGS). The 5STS and 30STS were performed using a standard chair (30). Participants crossed both arms against the chest, started from a seated position (upper back against seat), stood up to full extension, and sat down again (upper back against seat). The time required to perform 5 chair stands (until the fifth standing position) was evaluated in the 5STS. In the 30STS, the number of successful repetitions over a 30-second period was counted. The TUG was performed by standing up from a standard chair, walk a 3-m distance, turn, walk back, and sit down again as fast as possible without running (31). The MGS was calculated as the time needed to walk 10 m as fast as possible (without running). Time (seconds) was registered through timing gates (Racetime2 Light Radio, Microgate, Italy). HGS was measured using a Jamar hand dynamometer (Henrotech, Aartselaar, Belgium), which was adjusted for hand size. Handgrip strength was performed with the dominant hand, in a sitting position with the upper arm hanging by side and the elbow in 90° flexion. All tests were performed twice, except 30STS (once) and HGS (3 times). The best performance was used for further analysis.

#### Body composition

Parameters of body composition, that is, fat mass% and lean mass%, were calculated from the body’s impedance through bioelectrical impedance analysis (Bodystat 1500MDD, EuroMedix, Wijgmaal, Belgium).

#### Blood sample analyses

Fasted blood samples were collected from a cubital vein into vacutainers containing lithium heparin, EDTA, or Silica Clot Activator and centrifuged (1500 rpm for 15 min at 4°C), and the supernatant was stored at −20°C until later analysis. Plasma was analyzed for high-sensitive C-reactive protein (hsCRP; primary outcome), triglycerides, total cholesterol, high-density lipoprotein (HDL) cholesterol, and low-density lipoprotein (LDL) cholesterol (HITACHI/Roche COBAS 800 c502, Roche Diagnostics, Indianapolis, IN). Serum was analyzed for insulin (COBAS 8000, HITACHI/Roche, Tokyo, Japan) and interleukin (IL)-6 (primary outcome), in triplicate, with a commercially available ELISA (HS-600C, R&D, Minneapolis, MN). Fasted capillary blood samples from the earlobe were immediately analyzed for blood glucose levels (Glucomen Lx plus-meter with GlucoMen LX sensor strips, Menarini Diagnostics, Firenze, Italy). HOMA-IR was calculated as fasting insulin (mIU/L) × fasting glucose (mg/dL)/405.

### Statistical Analyses

Data are presented as means ± SEM. Data were tested for normality with a Kolmogorov–Smirnov test. Depending on the normality of the data, baseline differences between groups were analyzed using either independent samples *t* tests or Mann–Whitney *U* tests. To check for time and Condition*Time interaction effects, nonnormally distributed data (Kolmogorov–Smirnov *p* < .05) were first log-transformed and subsequently analyzed using a 2-way repeated measures ANOVA with time as within-subjects factor and group as between-subjects factor. To address sex-specific effects of ω-3 supplementation on isometric strength, a 3-way repeated measures ANOVA design was applied (Condition*Time*Sex). Accordingly, the muscle anabolic response was analyzed using a 3-way repeated measures ANOVA (Condition*Time*State [fasted vs fed]). Significant interaction effects are followed by a Bonferroni post hoc test. Significance was set at *p* <.05, trends were set at *p* = .05–.10. *p*-Values of log-transformed data are indicated with *p*^l^. Outliers were detected with Grubbs’ test at *p* <.05 (GraphPad Software, La Jolla, CA). Where applied, outlier exclusion is explicitly mentioned in the manuscript. Where relevant, eta-squared (η ^2^) was used as an index of effect size for ANOVA (.02 = small, .13 = medium, .26 = large).

## Results

### Blood Analyses

One subject was excluded from blood analyses due to a lacking blood sample (Supplementary Table 3). For the hsCRP analyses, one additional subject was not included due to a statistical outlier (10.59 mg/L; *p* < .05), probably related to an earlier acute infection and therefore not relevant in the context of low-grade, chronic inflammation. No interaction effects were observed for any of the variables. However, for IL-6, there was a trend towards an interaction effect (η ^2^ = .15; *p*^1^ = .074), with an increase in PLAC (+0.48 pg/mL) and a slight decrease in ω-3 (−0.21 pg/mL). Also for HOMA-IR, there was a trend for interaction (*p* = .087), indicating a decrease in PLAC (−10%) and an increase in ω-3 (+9%). There was a significant increase in HDL cholesterol over time (η ^2^ = .23; *p* = .02), that is, +3.7% in PLAC and +11.6% in ω-3. IL-6 and fasting glucose tended to increase over time, that is, +5% (η ^2^ = .15; *p*^1^ = .058) and +4% (η ^2^ = .15; *p*^1^ = .065), respectively.

### Muscle Strength

**Figure 1.**
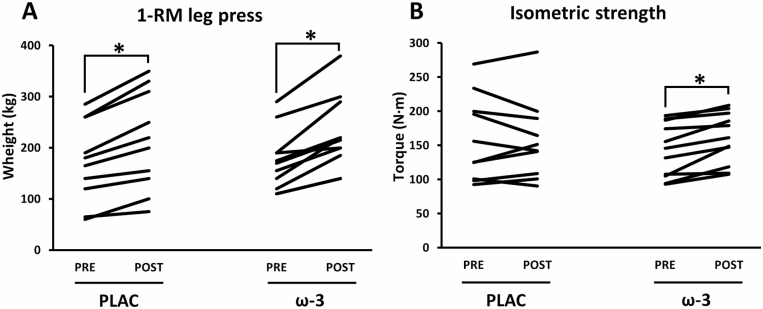
Muscle strength before (PRE) and after (POST) 12-week resistance exercise in the placebo (PLAC) and omega-3 (ω-3) group. (**A**) One-repetition maximum (1-RM) on the leg press device (ω-3: *n* = 11; PLAC: *n* = 10) and (**B**) isometric knee-extensor strength (90° knee angle) on the Biodex device (ω-3: *n* = 11; PLAC: *n* = 10). Data were analyzed with a 2-way repeated measures ANOVA to detect differences over time (PRE vs POST) and between groups (PLAC vs Ω-3). *p < .05 (POST vs PRE).

For maximal strength measures (leg press 1-RM and isometric strength), one participant was excluded due to low back pain with limited maximal force production during the POST-test (Figure 1). Similarly, for both maximal strength measures, another participant was excluded due to outlier statistics (leg press 1-RM gain of +108%; *p* < .05). This might be explained by a restrained 1-RM PRE-test due to anxiety for physical complications resulting from the maximal effort. Leg press 1-RM was significantly improved following RE in both conditions (η ^2^ = .80; *p* < .001), that is, an increase of +23.5% in PLAC (from 172.5 ± 25.0 to 213.0 ± 30.5 kg) and +30.4% in ω-3 (from 173.6 ± 17.6 to 226.4 ± 21.6 kg). There was no interaction effect (*p* = .27). Isometric muscle strength tended to increase over time (+5.4%; η ^2^ = .19; *p* = .052). There was also a significant interaction effect for isometric muscle strength, with an increase in ω-3 (+12.2%) and not in PLAC (−1.4%; η ^2^ = .27; *p* = .015).

### Leg Muscle Volume

The muscle volume did not significantly change due to the RE intervention in either the PLAC or the ω-3 group (Supplementary Table 4). For the analysis of the muscle HU values, one participant was removed from analysis due to outlier statistics (change in muscle HU of −10.1%; *p* < .05). Muscle HU significantly increased over time (+3.4%; η ^2^ = .50; *p* < .001). Calf circumference tended to increase over time from 37.3 ± 0.4 to 37.8 ± 0.5 cm (+1.3%; η ^2^ = .13; *p* = .09). There was no interaction effect for any of these parameters.

### Functional Performance

There was a significant time effect for 5STS (−13.7%; η ^2^ = .73), 30STS (+9.7%; η ^2^ = .52), and TUG (−6.3%; η ^2^ = .29), but not for MGS (−3.4%) and HGS (+2.7%). No interaction effect was observed for any of the performance tests (Supplementary Table 5).

### Body Composition

There was no significant change in BM (from 77.0 ± 2.2 to 77.4 ± 2.2 kg; *p* = .20), in fat mass% (from 31.5 ± 1.7 to 31.6 ± 1.7%; *p*^l^ = .76) or in lean mass% (from 68.4 ± 1.7 to 68.4 ± 1.7%; *p*^l^ = .89). There was no interaction effect for BM (*p* = .32) or fat mass% (*p*^l^ = .10). However, lean mass% tended to increase in the ω-3 group (+0.9%), while there was a decrease in the PLAC group (−1.1%; η ^2^ = .14; *p*^l^ = .08).

### Muscle Inflammation

**Figure 2.**
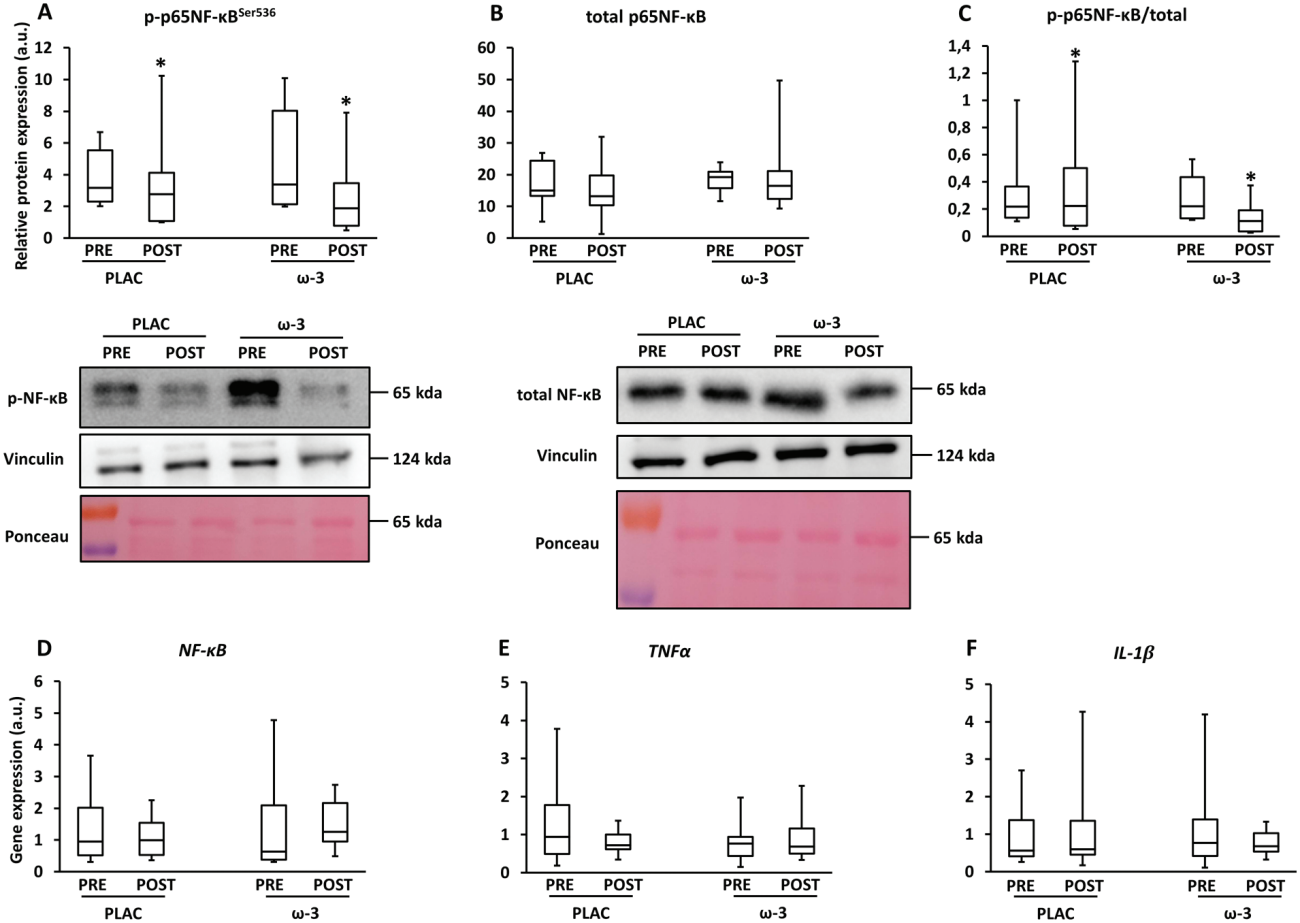
Expression of inflammatory markers in skeletal muscle tissue before (PRE) and after (POST) 12-week resistance exercise in the placebo (PLAC) and omega-3 (ω-3) group. (**A**) Phosphorylation of the p65 subunit of the NF-kappa-B transcription complex at the Ser536-site (p-p65NF-κB^Ser536^), (**B**) total p65NF-κB, (**C**) p-p65NF-κBSer536/total p65NF-κB, (**D**) NF-κB gene expression, (**E**) tumor necrosis factor alpha (TNFα) gene expression, (**F**) interleukin-1β (IL-1β) gene expression. For all boxplots, the box represents the interquartile range, the horizontal line in the box represents the median, and the error bars represent the maximum and minimum values. Data were analyzed with a 2-way repeated measures ANOVA to detect differences over time (PRE vs POST) and between groups (PLAC vs Ω-3). *p < .05 (POST vs PRE).

Expression of the key proinflammatory transcription factor phospho/total p65NFκB decreased over time (−19%; η ^2^ = .25; *p*^l^ = .030; Figure 2A–C). Furthermore, there was a trend for interaction (ie, +13% in PLAC and −52% in ω-3; η ^2^ = .15; *p*^l^ = .099). NF-κB (*p*^l^_time_ = .60; *p*^l^_interaction_ = .47), TNFα (*p*^l^_time_ = .75; *p*^l^_interaction_ = .37), and IL-1β (*p*^l^_time_ = .68; *p*^l^_interaction_ = .46) gene expression remained unchanged (Figure 2D–F).

### Muscle Anabolic Sensitivity

**Figure 3.**
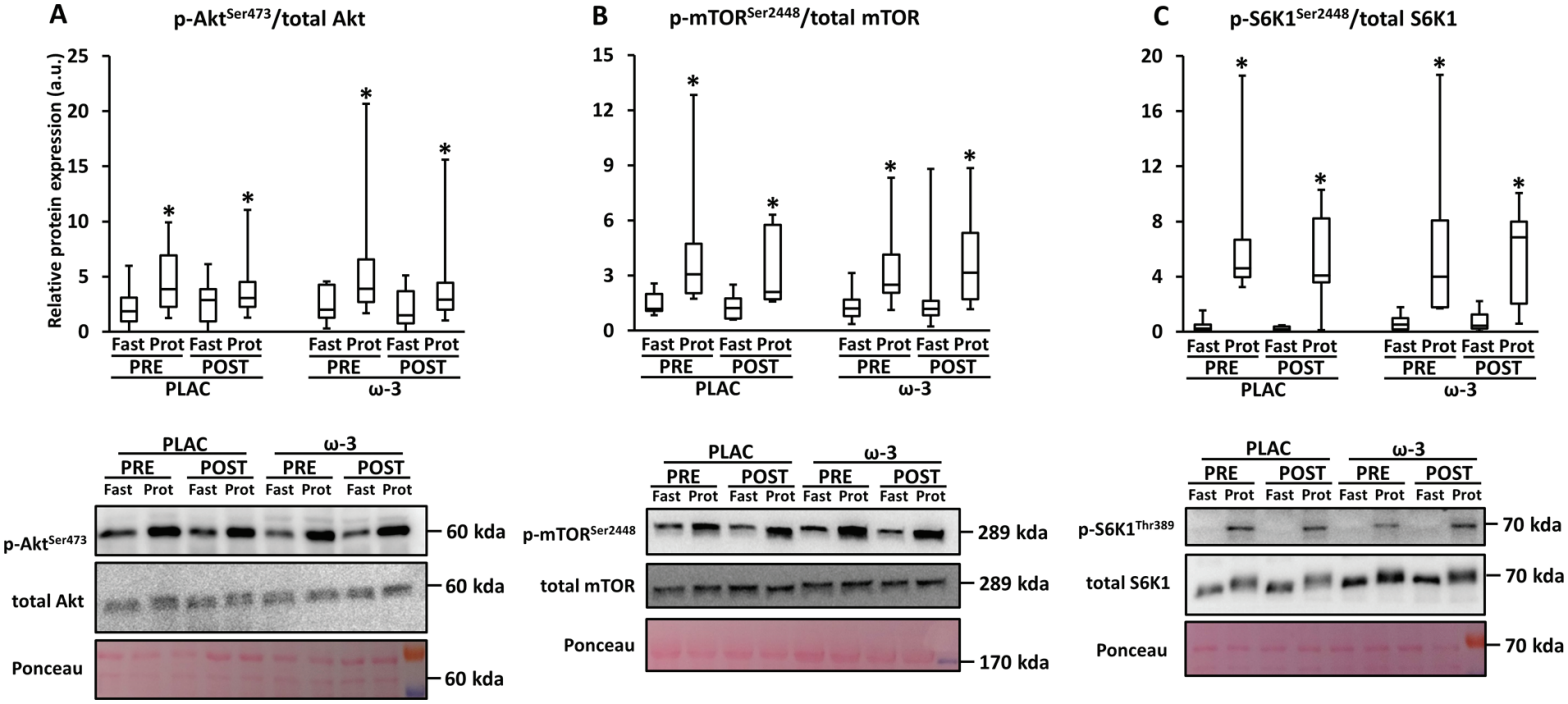
Expression of protein synthesis markers in skeletal muscle tissue in the fasted state (Fast) and after protein bolus (Prot), before (PRE) and after (POST) 12-week resistance exercise in the placebo (PLAC) and omega-3 (ω-3) group. (**A**) Phosphorylation of Akt at the Ser473-site/total Akt, (**B**) phosphorylation of mammalian target of rapamycin (mTOR) at the Ser2448-site/total mTOR, (**C**) phosphorylation of ribosomal S6 kinase 1 (S6K1)/total S6K. For all boxplots, the box represents the interquartile range, the horizontal line in the box represents the median, and the error bars represent the maximum and minimum values. Data were analyzed with a 3-way repeated measures ANOVA to detect differences over time (PRE vs POST), between groups (PLAC vs Ω-3), and state (fasted vs after protein bolus). *p < .05 (POST vs PRE).

Phospho/total protein expression ratio of Akt, mTOR and S6K1 significantly increased after breakfast versus fasted state (η ^2^ = .63–.89; *p*^l^ < .001; Figure 3A–C). There was no main effect of time or condition, nor an interaction effect for Time*Condition or Time*Condition*State. However, p-Akt/total Akt tended to be less increased (fed vs fasted) POST versus PRE, irrespective of condition (η ^2^ = .16; *p*^l^ = .094 for interaction Time*State).

### Muscle Catabolism

**Figure 4.**
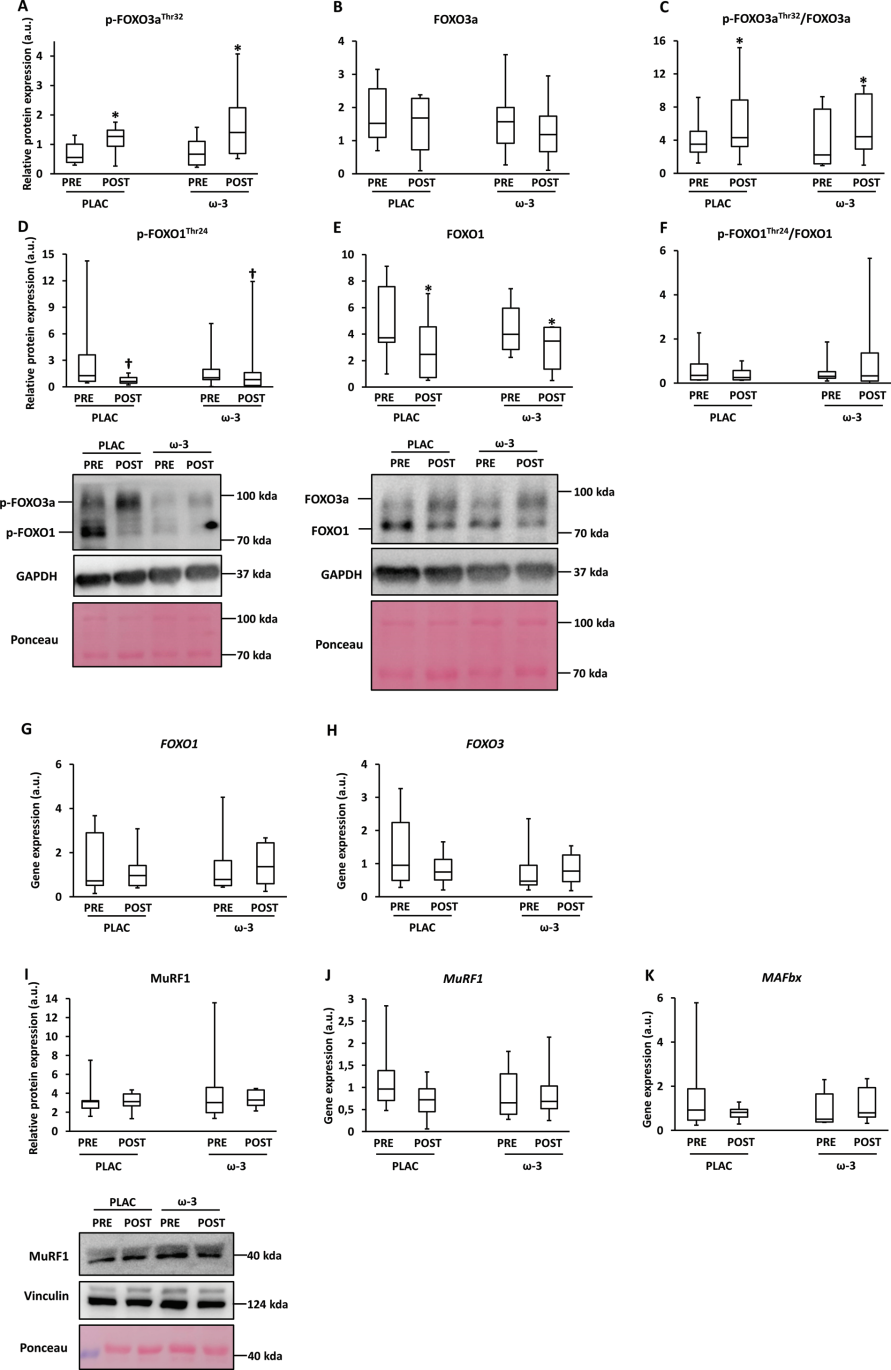
Expression of ubiquitin-proteasome markers in skeletal muscle tissue before (PRE) and after (POST) 12-week resistance exercise in the placebo (PLAC) and omega-3 (ω-3) group. (**A**) Phosphorylated forkhead box O (FOXO) 3a at the Thr32-site, (**B**) total FOXO3a, (**C**) p-FOXO3a/total FOXO3a, (**D**) p-FOXO1 at the Thr24-site, (**E**) total FOXO1, (**F**) p-FOXO1/total FOXO1, (**G**) *FOXO1* gene expression, (**H**) *FOXO3* gene expression, (**I**) muscle-specific RING finger protein 1 (MuRF1) protein expression, (**J**) *MuRF1* gene expression, (**K**) *muscle atrophy F-box* (*MAFbx*) gene expression. For all boxplots, the box represents the interquartile range, the horizontal line in the box represents the median, and the error bars represent the maximum and minimum values. Data were analyzed with a 2-way repeated measures ANOVA to detect differences over time (PRE vs POST) and between groups (PLAC vs Ω-3). *p < .05 (POST vs PRE); ^†^*p* = .5–.1 (POST vs PRE).

The UPS marker FOXO was affected by training, not by ω-3 supplementation. Phospho-FOXO3a was higher POST versus PRE, irrespective of condition (+87%; η ^2^ = .61; *p*^l^_time_ < .001; Figure 4A), whereas total FOXO3a was not affected (*p*^l^_time_ = .22; Figure 4B). This resulted in an increased FOXO3a phospho/total ratio (η ^2^ = .30; *p*^l^_time_ = .015; Figure 4C) and suggests an increase in the cytoplasmic (phospho) but not in the nuclear (nonphosphorylated) abundance of FOXO3a. Phospho-FOXO1 tended to be lower at POST versus PRE (−41%; η ^2^ = .18; *p*^l^ = .059; Figure 4D), whereas total FOXO1 was significantly lower at POST versus PRE (−36%; η ^2^ = .31; *p* = .028; Figure 4E). The FOXO1 phospho/total ratio remained unchanged (*p*^l^_time_ = .54; Figure 4F). FOXO1 and FOXO3 gene expression were unaltered (*p*_time_ = .38 and .15, respectively; Figure 4G and H). Protein levels (*p*^l^_time_ = .90; *p*^l^_interaction_ = .97) and gene expression (*p*^l^_time_ = .74; *p*^l^_interaction_ = .12) of the E3 ubiquitin ligase MuRF1, and MAFbx (*p*_time_ = .14; *p*_interaction_ = .16) gene expression were unaffected by time or condition (Figure 4I–K).

**Figure 5.**
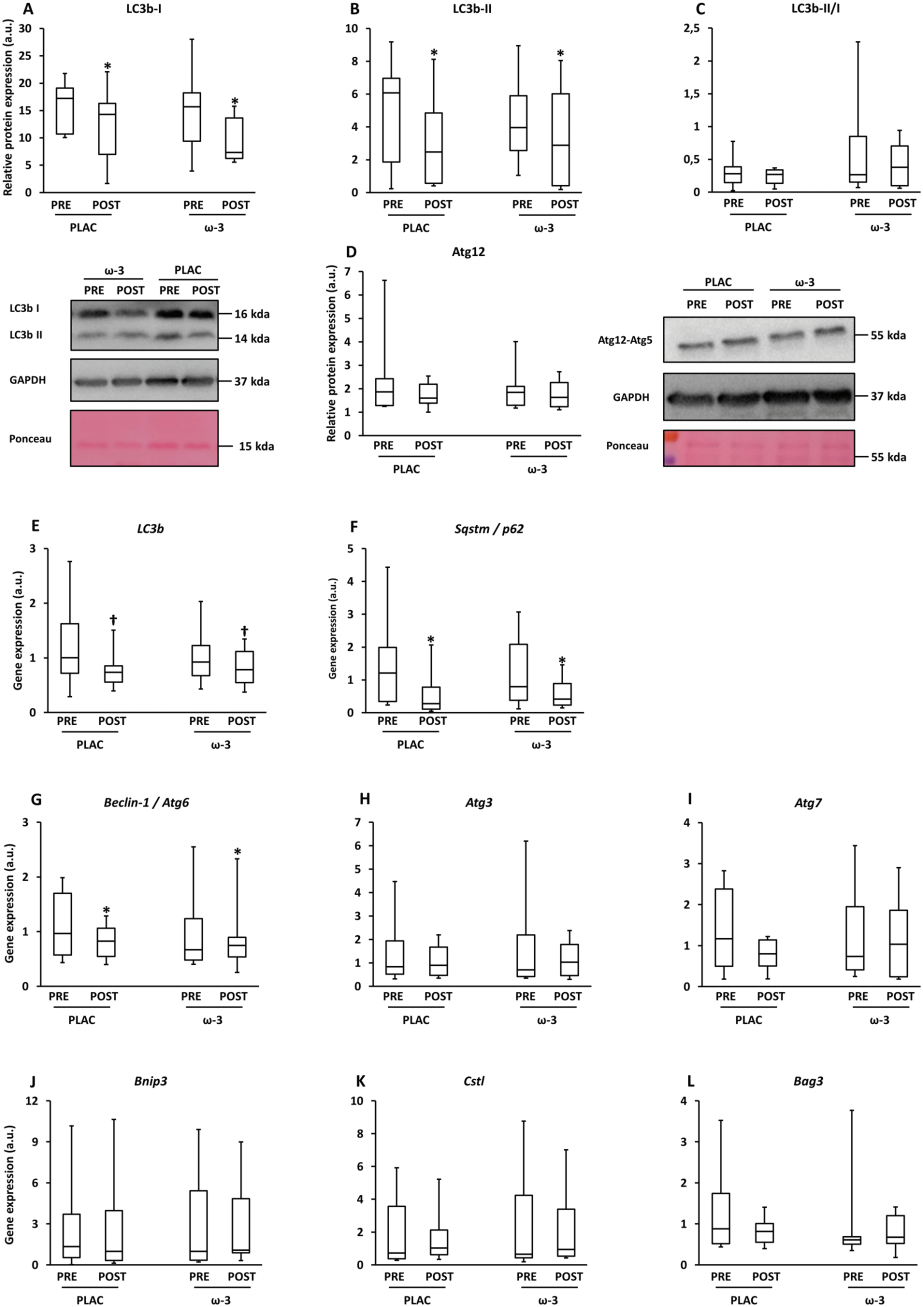
Expression of autophagy markers in skeletal muscle tissue before (PRE) and after (POST) 12-week resistance exercise in the placebo (PLAC) and omega-3 (ω-3) group. (**A**) Microtubule-associated proteins 1a/1b light chain 3b (LC3b) I protein expression, (**B**) LC3b-II protein expression, (**C**) LC3b II/LC3b I, (**D**) autophagy-related protein (Atg)5-Atg12 expression, (**E**) *LC3b* gene expression, (**F**) *sequestosome-1* (*Sqstm1*) / *p62* gene expression, (**G**) *Beclin-1* / *Atg6* gene expression, (**H**) *Atg3* gene expression, (**I**) *Atg7* gene expression, (**J**) *Bnip3* gene expression, (**K**) *Cathepsin L* (*Cstl*) gene expression, (**L**) *B-cell lymphoma 2–associated anthanogene* (*Bag3*) gene expression. For all boxplots, the box represents the interquartile range, the horizontal line in the box represents the median, and the error bars represent the maximum and minimum values. Data were analyzed with a 2-way repeated measures ANOVA to detect differences over time (PRE vs POST) and between groups (PLAC vs Ω-3). *p < .05 (POST vs PRE); ^†^*p* = .5–.1 (POST vs PRE).

The autophagy marker LC3b comprises of LC3b-I, which is found in the cytoplasm, and LC3-II, which is membrane-bound and is converted from LC3-I to initiate formation and lengthening of the autophagosome. Both forms were lower at POST versus PRE intervention (LC3b-I: −28%, η ^2^ = .26, *p*_time_ = .029; LC3b-II: −28%, η ^2^ = .22, *p*^l^_time_ = .042), but unaltered due to ω-3 (*p*_interaction_ = .48 and .84, respectively; Figure 5A and B). LC3b-II/I was unaltered by time or condition (*p*^l^_time_ = .51; *p*^l^_interaction_ = .75; Figure 5C). LC3b gene expression tended to be lower at POST versus PRE intervention (−27%; η ^2^ = .19; *p*_time_ = .057), irrespective of condition (*p*_interaction_ = .44; Figure 5E). Similarly, autophagy markers Sqstm1/p62 and Beclin-1 were lower at POST versus PRE (−55%; η ^2^ = .32; *p*^l^_time_ = .009 and −18%; η ^2^ = .21; *p*^l^_time_ = .043, respectively; Figure 5F and G). Yet, protein expression of Atg5-Atg12 (*p*_time_ = .19; *p*_interaction_ = .48; Figure 5D) and gene expression of Bnip3 (*p*^l^_time_ = .63; *p*^l^_interaction_ = .85), Atg3 (*p*^l^_time_ = .65; *p*^l^_interaction_ = .97), Atg7 (*p*^l^_time_ = .36; *p*^l^_interaction_ = .54), Cstl (*p*^l^_time_ = .57; *p*^l^_interaction_ = .83), and Bag3 (*p*^l^_time_ = .40; *p*^l^_interaction_ = .40) (Figure 5G–L) were unaffected.

## Discussion

We hypothesized that ω-3 supplementation would enhance the muscular responses to RE, both on a functional and molecular level, in healthy OA. However, besides additional gains in isometric strength, ω-3 did not improve muscle volume, functionality, or inflammatory, anabolic or catabolic signaling upon RE. Our data confirm that RE is key for beneficial adaptations in skeletal muscle. Besides improved strength, RE was effective to downregulate markers of muscle inflammation and catabolism.

Ω-3 supplementation has been shown to reduce systemic levels of proinflammatory markers, such as CRP, IL-6, and TNFα, in overweight (14) and obese (15) adults, and in patient populations (16). However, the anti-inflammatory effect of ω-3 was not consistently demonstrated in healthy (middle-)aged adults (50–94 years) (17–19). Similar to our study, Da Boit et al. (18 weeks) and Cornish et al. (12 weeks) combined ω-3 supplementation (3 g/d) with RE in healthy OA (~70 years) (18,19). They found that the systemic IL-6 and TNFα concentrations remained unchanged by ω-3 supplementation. It can be hypothesized that the lack of anti-inflammatory effects due to ω-3 might be partly attributed to a low baseline inflammation (hsCRP of 1.05 ± 0.23 mg/L), leaving very little room for improvement. However, in the present study, even in healthy OA with low baseline inflammation, ω-3 decreased hsCRP by 30%, or 0.33 mg/L. Despite being a potentially clinically relevant decrease, significance was not reached because of a high variation in hsCRP between subjects (range: 0.15–4.79 mg/L). Besides, it is conceivable that beneficial muscular adaptations due to ω-3 are related to direct anti-inflammatory mechanisms in the skeletal muscle. Surprisingly, no studies have tested this hypothesis.

The present study is the first to address the effects of ω-3 on muscle inflammation in OA (~70 years). We observed a tendency towards a decreased p-p65NF-κB expression due to ω-3 (Figure 2C), but no changes in TNFα and IL-1β (Figure 2E,F). Few studies in mice looked at the effect of ω-3 on muscle inflammation. In a murine atrophy model of Duchenne muscular disease (mdx), ω-3 treatment decreased the TNFα but not NF-κB muscle expression (32). Similarly, muscle catabolism (UPS and autophagy) was unaffected by ω-3 (Figure 4,5). There are very few data concerning ω-3-induced effects on catabolism in humans. Microarray analyses revealed that 6-month ω-3 supplementation (3.4 g/d) downregulated ubiquitin-mediated proteolysis in OAs (~70 years) (33). However, this was not confirmed by gene expression data of UPS (*FOXO3*, *MuRF1*, *MAFbx*) and autophagy markers (*LC3*, *GABARAP*) (33). In murine atrophy models (starvation (34), arthritis (35), sepsis (21), cachexia (22), immobilization (36)), ω-3 decreased UPS markers and thereby prevented muscle loss. However, this was not confirmed in murine (37) and human (38) immobilization studies.

In contrast to ω-3 supplementation, the effect of RE on muscle inflammation is better described in humans. We observed a decreased p-p65NF-κB expression upon RE, whereas other proinflammatory mediators (IL-1β, TNFα) remained unaffected (Figure 2). Similarly, 12-week (39) or 12-month (40) RE training did not affect muscle inflammation (eg, IL-6, TNFα, IL-8, MCP-1) in healthy OAs (60–75 years). In contrast, Greiwe et al. found that a 12-week RE program decreased muscle TNFα levels in OA(~80 years) (41). This difference could be attributed to the age difference (~80 years in the study of Greiwe et al. (41) versus ~70 years in the study of Della Gatta et al. (39), Ziegler et al. (40), and in the present study). At the age of 80 years, it is more likely that baseline muscle inflammation is more pronounced (42). To fully uncover the muscle anti-inflammatory potential of RE, it is probably more effective to include a frail, old population that suffers from elevated proinflammatory muscle signatures at baseline.

Interestingly, we observed significant decreases in several autophagy markers in the skeletal muscle following of RE training, that is, LC3b-I and LC3b-II protein expression, and Sqstm and beclin-1 gene expression (Figure 5). Earlier evidence concerning the role of RE on muscle autophagy is ambiguous. RE upregulated LC3b-II, downregulated p-ULK1^Ser555^ and beclin-2, and did not affect beclin-1 or p62 expression in young adults but not OAs (61 years) (43). It should be noted that Hentilä et al. (43) observed a tendency towards decreased expression POST versus PRE for beclin-1 (−20%), beclin-2 (−0%), and p62 (−38%) in OA, but this did not result in significance due to limited sample size (*n* = 6) and high variability among participants.

Whether decreased muscle autophagy is beneficial for healthy muscle ageing remains a matter of debate. Very low and very high muscle autophagy are deleterious for muscle homeostasis due to insufficient clearance of intracellular waste products and an increased breakdown of functional and/or structural muscle proteins, respectively. Autophagy markers LC3b-II (44), Atg7 (45), and beclin-1 (45) were increased in skeletal muscle of OAs (70–88 years) compared to young adults. Given that RE downregulates autophagy markers in old skeletal muscle and that this downregulation is accompanied by beneficial muscular adaptations (eg, strength), it is very likely that the decreased autophagy is still sufficient to exert housekeeping functioning, for example, clearance of damaged cell organelles. Hence, part of the beneficial muscular adaptations upon RE in healthy OAs can be ascribed to a decreased, yet sufficiently high, muscle autophagy.

The potential of ω-3 to improve muscle health in muscle devastating conditions such as sarcopenia and cachexia is mainly based on their capacity to stimulate muscle anabolism. Smith et al. found that 8-week ω-3 supplementation (3.4 g/d) upregulated mTORC1 signaling and muscle protein synthesis in healthy OAs (~71 years) (20). Microarray data from the same group revealed that this was (partly) due to a downregulation of mTOR inhibition pathways (33). Our data revealed no effect of ω-3 supplementation on key markers of the mTORC1 pathway (Figure 3). Similarly, Da Boit et al. found that 18-week ω-3 supplementation (3 g/d) did not alter muscle S6K1 activity or muscle protein synthesis near the end of a RE protocol compared to safflower-treated OA (~70 years) (18). Discrepancies in the effect of ω-3 on mTORC1 stimulation between studies can be attributed to methodological issues, such as mTORC1 stimulus (Smith et al. (20): hyperaminoacidemic–hyperinsulinemic clamp vs Da Boit et al. (18): acute RE vs the present study: a protein-rich meal) and study intervention (the present study and that of Da Boit et al. (18): RE vs the study of Smith et al. (20): no RE). Whereas 12-week RE in OAs (~71 years) did not affect mTORC1 signaling in response to an amino acid bolus (46), this does not exclude that RE might interfere with the effect(s) of ω-3 on muscle mTORC1 signaling. However, this is rather speculative as a physiological mechanism supporting this hypothesis is currently lacking.

The mechanisms via which anabolic insensitivity develops with ageing are not fully understood. One hypothesis states that “inflamm-ageing” might contribute to muscle anabolic resistance in OA. Hence, the lack of a straightforward anti-inflammatory effect due to ω-3 in the present study might explain why mTORC1 was not upregulated upon ω-3 supplementation in the present study. However, Smith et al. observed that the clamp-induced mTORC1 upregulation following ω-3 supplementation in OAs occurred without any changes in systemic hsCRP, IL-6 or TNFα (20). Therefore, there are other mechanisms to explain the anabolic stimulatory effect of ω-3. Microarray data suggested that ω-3 supplementation in OA downregulates mTORC1 inhibitors (33). However, this was not confirmed by the muscle gene expression of key regulators of hypertrophy, atrophy, and regeneration (33). Furthermore, no other studies have reported any link between ω-3 and muscle mTORC1 inhibitors. Hence, future studies should confirm on gene expression and/or protein levels whether ω-3 downregulates mTORC1 inhibitors in muscle.

Besides molecular outcomes, this study aimed to investigate whether ω-3 can improve functional adaptations to RE in healthy OAs. Interestingly, we found that ω-3 increased the isometric knee-extensor strength (+12.2%) in response to RE, while this was not the case in the control group (−1.4%). Accordingly, others found that ω-3 supplementation and RE increased isometric knee-extensor strength to a higher extent than RE controls (no ω-3) in older women (18,47). However, these beneficial strength adaptations due to ω-3 were not present in older men (~70 years) (18), suggesting a sex-specific effect of ω-3 on isometric strength. Our data do not support this sex specificity. For both sexes, the ω-3 group had higher increases in isometric strength (♀: +21%; ♂: +9.2%) compared to PLAC (♀: +2.8%; ♂: −2.3%). A 3-way ANOVA (Condition*Time*Sex) revealed a Time*Condition (*p* = .032), but no Time*Sex or Time*Condition*Sex interaction. Our study comprises only 4 women per condition, thus conclusions should be treated with caution. The higher increase in isometric strength in the ω-3 group occurred in the absence of molecular or muscle volume adaptations due to ω-3. It has been suggested that ω-3 might increase the contractility in response to acetylcholine and prostaglandins, and/or that a greater membrane fluidity due to ω-3 may facilitate the neuromuscular pulse transmission (47,48). Although being intriguing hypotheses, future studies should confirm whether these mechanisms can explain the beneficial isometric strength response due to ω-3, while at the same time 1-RM leg press remained unaffected by ω-3.

In contrast to isometric strength, ω-3 did not increase the leg press 1-RM upon RE, which is in line with Cornish et al. (19). Different hypotheses might explain why ω-3 improves isometric but not leg press strength. Firstly, participants were trained thrice weekly for 12 weeks and during each session they performed multiple (30–45) repetitions. This might have plateaued the leg press strength, hence, leaving little additional room for improvement due to ω-3. Alternatively, the leg press is a multi-joint movement, which requires intermuscular coordination and therefore more neural control than the isometric test (single joint). Considering that the same leg press equipment was used for training and strength assessment, this neural component was extensively trained and might have masked potential differential (muscular) gains in both groups. Therefore, isometric strength might be a better representation of isolated muscular strength. In accordance with others, RE improved functional performance (18,19,47) and 1-RM (18,19) irrespective of ω-3, whereas isometric strength was further improved by ω-3 (18,47). The data of the present study indicate that beneficial effects upon RE due to ω-3 can mainly be attributed to muscular (structural, metabolic) and not neural adaptations.

Decreases in catabolic and inflammatory markers due to RE (eg, LC3b, FOXO1, p-p65NFκB) were not sufficient to affect muscle volume following 12 weeks RE. The lack of change in muscle volume due to RE and ω-3 is in accordance with Da Boit et al., who observed no changes in the quadriceps cross-sectional area upon a 18-week RE program with (3 g/d) or without ω-3 supplementation in OAs (18). This could be attributed to the duration of the intervention (12–18 weeks) or the ω-3 dose (~3 g/d). Indeed, a daily dose of 4g ω-3 during 16 weeks increased the total lean mass (+1.4%) in OAs (65–85 years) (49). Recently, it was shown in old mice that a 12-week ω-3 treatment improved muscle strength without changes in muscle mass (48). The authors suggested that the increase in muscle strength can be explained by beneficial effects of ω-3 on muscle quality.

There are still some crucial points concerning ω-3 supplementation that require further investigation to better understand differences in responses. First, it remains to be studied which biological mechanism(s) underlie possible sex-specific effects of ω-3. Furthermore, the baseline ω-3 intake and ω-3/ω-6 ratio of participants should be considered since simultaneous ω-6 membrane incorporation antagonizes ω-3-related health effects (50). It has also been suggested that (epi)genetic variability in genes encoding enzymes of ω-3 synthetic pathways and in microbiota background interfere with responses to ω-3 supplementation (50). It should also be noted that the large age span among study participants (65–84 years) might be a limitation of this study. Although we recruited a homogenous population in terms of physical activity, muscle strength, and inflammation, a 20-year age range might interfere with the systemic and muscular responses to the study intervention. Yet, there was no difference in baseline 1-RM or in the %increase in 1-RM between ≤70 years (176 kg, +27%) and >70 years (160 kg, +31%; *p* = .59 and *p* = .61, respectively). Finally, we hypothesized to counteract the age-related anabolic resistance. However, to determine whether and to which extent the participants suffered from anabolic resistance, a young control group should have been included in the study.

In conclusion, the present study shows that ω-3 supplementation improves isometric strength but not leg press 1-RM upon RE in healthy OA. Muscle anabolism, catabolism, and inflammation were not affected by ω-3. However, RE downregulated markers of muscle autophagy and the UPS, as well as muscle inflammation, but did not upregulate muscle anabolism. This study confirms RE to be key for beneficial muscular adaptations on a molecular and a functional level. The effect of ω-3 on muscular adaptations was limited, but might be more pronounced in frail OAs suffering from high systemic and/or muscular inflammation.

## Supplementary Material

glaa309_suppl_Supplementarty_Material
