# Supplementary material for: Omega-3 Supplementation Improves Isometric Strength But Not Muscle Anabolic and Catabolic Signaling in Response to Resistance Exercise in Healthy Older Adults
Source: J Gerontol A Biol Sci Med Sci. 2020 Dec 7;76(3):406–14. doi: 10.1093/gerona/glaa309 (PMC7907485; doi:10.1093/gerona/glaa309)
Supplement: glaa309_suppl_Supplementarty_Material [file glaa309_suppl_supplementarty_material.pdf]

Omega-3 supplementation improves isometric strength but not muscle anabolic and catabolic signaling in response to resistance exercise in healthy older adults

Online-Only Supplemental Material

Index

Methods: RNA extraction, reverse transcription and real-time quantitative PCR analyses

Methods: Protein extraction and western blot analyses

eTable 1. Baseline characteristics of the study participants.

eTable 2. Primer sequences used for real-time PCR.

eTable 3 (n=9-11/condition). Plasma levels of hsCRP, triglycerides, HDL, LDL and total cholesterol, fasting insulin and fasting glucose, and HOMA-IR in participants supplemented with either omega-3 (ω-3) or corn oil (PLAC) before (PRE) and after (POST) 12-wk resistance exercise.

eTable 4 (n=11-12/condition). Upper leg muscle volume (cm^3), muscle density (HU) and muscle contribution (muscle volume / total volume, %) in participants supplemented with either omega-3 (ω-3) or corn oil (PLAC) before (PRE) and after (POST) 12-wk resistance exercise.

eTable 5 (n=11-12/condition). A 5-repetition chair sit-to-stand test (5STS), 30-second chair sit-to-stand test (30STS), timed up-and-go test (TUG), maximal gait speed test (MGS) and handgrip strength test (HGS) in participants supplemented with either omega-3 (ω-3) or corn oil (PLAC) before (PRE) and after (POST) 12-wk resistance exercise.

RNA extraction, reverse transcription and real-time quantitative PCR analyses

Total RNA was extracted from ~15mg frozen muscle tissue using TRI Reagent (Molecular Research Center, Cincinnati, OH). RNA quality and quantity was assessed with the SimpliNano spectrophotometer (GE Healthcare, Chicago, IL). cDNA was synthesized from 0.9µg RNA using the cDNA Reverse Transcription kit (Qiagen, Hilden, Germany). Real-time quantitative polymerase chain reaction (PCR) was performed using the GoTaq qPCR Master Mix (Promega, Madison, WI) on a QuantStudio 3 thermocycler (Thermo Fisher Scientific, Waltham, MA). Real-time PCR primers were designed for human NF-κB1, IL-1β, TNFα, FOXO1, FOXO3, F-box protein 32 (Fbxo32, MAFbx), Trim63 (MuRF1), Sqstm1 (p62), Beclin1, Map1lc3a (Lc3b), Atg3, Atg7, Bnip3, Cstl, Bag3 and GAPDH (eTable 2). Gene expression was calculated using the delta delta threshold cycle method with GAPDH as a reference gene.

Protein extraction and western blot analyses

Frozen muscle tissue (15-20mg) was homogenized 4 x 20s in ice-cold lysis buffer (1:10 w/v; 50mM Tris-HCl, pH 7.0; 270mM sucrose; 5mM EGTA; 1mM EDTA; 1mM sodium orthovanadate; 50mM glycerophosphate; 5mM sodium pyrophosphate; 50mM sodium fluoride; 1mM dithiothreitol; 0.1% Triton X-100; and a complete protease inhibitor tablet [Roche Applied Science, Vilvoorde, Belgium]) using the FastPrep (MP Biomedicals, Santa Ana, CA). Homogenates were then centrifuged (10 000g for 20min at 4°C and the supernatant was stored at -80°C. Protein concentrations were assessed with the DC protein assay kit applying a BSA protein standard (Bio-Rad Laboratories, Nazareth, Belgium). Lysis buffer was added to equalize protein concentrations. Eventually, laemmli (20% of the total volume) was added to obtain muscle lysates.

Proteins (20-30µg) were separated by SDS-PAGE (8-15% gels) and transferred to polyvinylidene difluoride membranes. Hereafter, membranes were blocked in TBS-T (tris-buffered saline with Tween-20) containing 5% non-fat milk or BSA for 1h. Next, membranes were incubated overnight at 4°C in TBS-T containing 5% non-fat milk or 5% BSA with the following antibodies: p-nuclear factor kappa B (p-p65NF-κB, sc-136548, 1/500), NF-κB (sc-372, 1/1000) and muscle RING finger-1 (MuRF1, sc-2920, 1/500) from Santa Cruz Biotechnology (Dallas, TX); p-Akt (CST-4058S, 1/1000), Akt (CST-2920, 1/1000), p-mammalian target of rapamycin (p-mTOR, CST-2971S, 1/1000), mTOR (CST-2983S, 1/1000), ribosomal protein S6 kinase 1 (S6K1, CST-9206S, 1/1000), glyceraldehyde 3-phosphate dehydrogenase (GAPDH, CST-2118S, 1/5000), p-forkhead box O (FOXO) 1/3a (CST-9464S, 1/500) and FOXO3a (CST-2497S, 1/500) from Cell Signaling (Leiden, The Netherlands); Vinculin (V9131, 1/1000) from Sigma Aldrich (Bornem, Belgium). Membranes were then incubated for 45min at room temperature with the appropriate horseradish peroxidase-conjugated secondary antibodies (1/7000; Sigma Aldrich, Bornem, Belgium). Membranes were scanned and quantified with Genesnap and Genetools Softwares (Syngene, Cambridge, UK). Results are presented as the protein of interest, relative to housekeeping proteins GAPDH or vinculin, and/or as the ratio phosphorylated/total form.

eTable 3. Baseline characteristics of the study participants.

	PLAC	Ω-3	p-value
**Sex (#)**	4 ♀ / 7 ♂	4♀ / 8 ♂	
**Age (y)**	70.6 ± 1.5	71.2 ± 1.0	0.77
**Weight (kg)**	77.0 ± 3.3	77.0 ± 3.0	0.99
**Height (cm)**	169.6 ± 2.7	168.4 ± 2.1	0.49
**BMI ($kg \cdot m^{-2}$)**	26.7 ± 0.4	27.1 ± 0.7	0.70

Data are presented as mean ± SEM. Baseline differences between groups were analysed with either independent samples t-tests (Age, Weight, BMI) or Mann-Whitney U tests (Height).

eTable 4. Primer sequences used for real-time PCR.

Gene	Forward	Reverse	Template	Product size
NF-κB	CCCAGTGAAGACCACCTCTC	AGCTCGTCTATTTGCTGCCT	NM_003998.4	71
IL-1β	AGCTGGAGAGTGTAGATCCCAAA	TCTGCTTGAGAGGTGCTGATG	NM_000576.3	142
TNFα	GCCCATGTTGTAGCAAACCC	TATCTCTCAGCTCCACGCCA	NM_000594.4	97
FOXO1	CAGAATGGGCCTTCTCCACC	AGTGTAACCTGCTCACTAACCC	NM_002015.4	227
FOXO3	TTGTTGGTTTGAACGTGGGG	TGTCAGTTTGAGGGTCTGCT	NM_001455.4	120
Fbxo32	CCGTCTCCCCATCCGTCT	AACTGCCGCTCTTCTCATCC	NM_058229.4	132
Trim63	TGCTCCATGTGCAAGGTGTTT	TTATTCAGTTCAGTCTTTTGTCCCT	NM_032588.3	92
Sqstm1	CTGAGGCGGAAGCCGAG	CCTCGTCACTGGAAAAGCCA	NM_003900.5	153
Beclin1	GAGGTGAAGAGCATCGGGGG	GCTGTGGTAAGTAATGGAGCTGTG	NM_003766.4	176
Map1lc3a	CTCAGACCGGCCTTTCAAGCA	GGGAGGCGTAGACCATATAGAGGA	NM_032514.4	341
Atg3	ACATGGCAATGGGCTACAGG	AGCACGGCACATTTTTGGTT	NM_022488.5	91
Atg7	GAGACCTGTATGTCCTGCGT	TGGTGTCCATCAGCTTCAGTTT	NM_001349232.2	110
Bnip3	AGCAATAATGGGAACGGGGG	TCCTCAGACTGTGAGCTGTTT	NM_004052.3	221
Cstl	CAAGTGGAAGGCTGCAATGGT	CCTTCTCCTGCTTAGGGATGT	NM_001257973.2	181
Bag3	GCTGCCTCTGACTGCTCATCC	GTGGGTCTGGTACTCCCCCT	NM_004281.4	207
GAPDH	TCGGAGTCAACGGATTTGG	TCGCCCCACTTGATTTTGGA	NM_002046.7	250

Atg3: autophagy-related 3; Bag3: B-cell lymphoma 2–associated anthanogene; Cstl: cathepsin L; Fbxo32: F-box protein 32; FOXO: forkhead box O; GAPDH: glyceraldehyde 3-phosphate dehydrogenase; IL-1β: interleukin 1 beta; Map1lc3a: microtubule-associated proteins 1A/1B light chain 3A; NF-κB: nuclear factor kappa B; Sqstm1: sequestosome-1; TNFα: tumour necrosis factor alpha

eTable 3 (n=9-11/condition). Plasma levels of hsCRP, triglycerides, HDL, LDL and total cholesterol, fasting insulin and fasting glucose, and HOMA-IR in participants supplemented with either omega-3 (ω-3) or corn oil (PLAC) before (PRE) and after (POST) 12-wk resistance exercise.

	PLAC		Ω-3		p-value	
	PRE	POST	PRE	POST	Time	Interaction
**hsCRP (mg·L^{-1}) [l]**	0.87±0.25	0.93±0.30	1.14±0.40	0.81±0.19	0.65	0.37
**IL-6 (pg·mL^{-1}) [l]**	2.93±0.99	3.41±1.18	2.11±0.41	1.90±0.31	0.06	0.07
**Triglycerides (mg·dL^{-1}) [l]**	142.6±12.4	107.4±11.4	126.3±28.9	109.2±12.9	0.35	0.99
**Total cholesterol (mg·dL^{-1})**	197.5±13.7	196.4±11.3	181.3±12.6	183.5±12.8	0.93	0.78
**HDL (mg·dL^{-1})**	54.6±4.4	56.6±3.5	50.3±2.3	56.1±3.4	0.02 *	0.25
**LDL (mg·dL^{-1})**	120.3±11.6	118.1±10.3	105.6±8.5	105.6±10.3	0.81	0.83
**Fasting insulin (mIU·L^{-1})**	8.7±1.0	8.0±0.8	9.1±1.2	9.4±0.9	0.73	0.43
**Fasting glucose (mg·dL^{-1}) [l]**	110.1±2.8	111.2±3.2	106.9±2.8	114.1±3.2	0.07	0.17
**HOMA-IR**	2.4±0.2	2.2±0.3	2.4±0.3	2.6±0.3	0.85	0.09

Data are presented as mean ± SEM. [l]: log-transformed data. Data were analysed with a 2-way repeated measures ANOVA to detect differences over time (PRE vs. POST) and between groups (PLAC vs. Ω-3). *$p < 0.05$. HDL: high-density lipoprotein; HOMA-IR: homeostatic model assessment of insulin resistance; LDL: low-density lipoprotein.

eTable 4 (n=11-12/condition). Upper leg muscle volume (cm^3), muscle density (HU) and muscle contribution (muscle volume / total volume, %) in participants supplemented with either omega-3 (ω-3) or corn oil (PLAC) before (PRE) and after (POST) 12-wk resistance exercise.

	PLAC		Ω-3		p-value	
	**PRE**	**POST**	**PRE**	**POST**	**Time**	**Interaction**
**Muscle volume (cm^3) [l]**	515.9±22.2	521.0±42.4	522.9±35.7	524.8±36.0	0.50	0.65
**Muscle density (HU)**	50.2±1.0	51.6±0.7	48.3±0.9	50.3±0.7	0.0002 *	0.45
**Muscle Contribution (%) [l]**	64.7±4.4	64.4 ± 4.3	65.3±3.4	64.8±3.4	0.42	0.77

Data are presented as mean ± SEM. [l]: log-transformed data. Data were analysed with a 2-way repeated measures ANOVA to detect differences over time (PRE vs. POST) and between groups (PLAC vs. Ω-3). *$p < 0.05$. HU: Hounsfield units.

eTable 5 (n=11-12/condition). A 5-repetition chair sit-to-stand test (5STS), 30-second chair sit-to-stand test (30STS), timed up-and-go test (TUG), maximal gait speed test (MGS) and handgrip strength test (HGS) in participants supplemented with either omega-3 (ω-3) or corn oil (PLAC) before (PRE) and after (POST) 12-wk resistance exercise.

	PLAC		Ω-3		p-value	
	**PRE**	**POST**	**PRE**	**POST**	**Time**	**Interaction**
**5STS (sec)**	8.8±0.2	7.5±0.2	8.4 ± 0.3	7.4±0.3	<0.001 *	0.27
**30STS (#)** [l]	16.7±0.5	18.3±0.4	16.6±0.6	18.3±0.8	<0.001 *	0.94
**TUG (sec)** [l]	5.8±0.3	5.6±0.2	6.3±0.2	5.8±0.2	0.02 *	0.23
**MGS (sec)**	4.8±0.3	4.6±0.2	5.0±0.2	4.8±0.2	0.15	0.93
**HGS (kg)**	33.9±3.8	34.8±3.9	32.7±2.9	33.6±3.3	0.31	1.00

Data are presented as mean ± SEM. [l]: log-transformed data. Data were analysed with a 2-way repeated measures ANOVA to detect differences over time (PRE vs. POST) and between groups (PLAC vs. Ω-3). *$p < 0.05$. 30STS: 30-second chair; 5STS: 5-repetition chair sit-to-stand; HGS: handgrip strength; MGS: maximal gait speed; TUG: timed up-and-go
